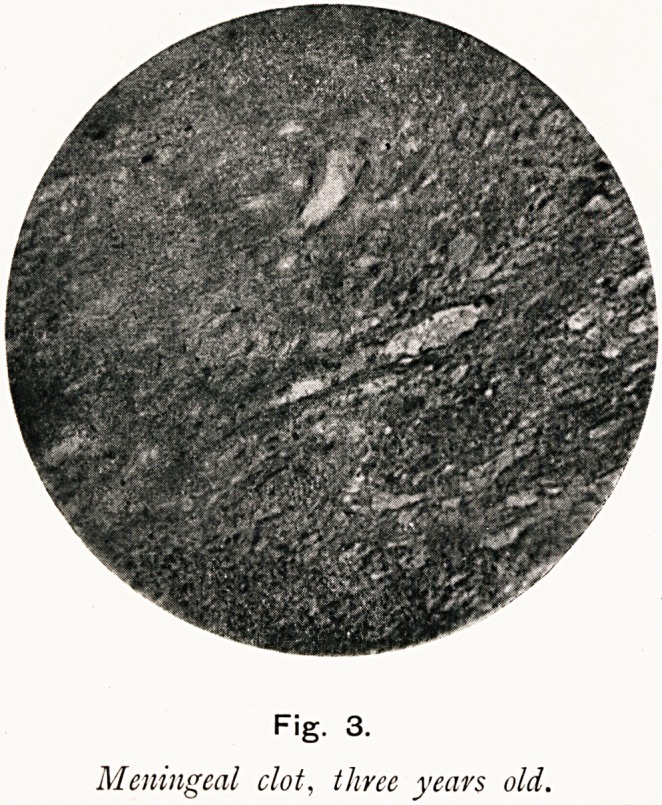# Notes on Three Pathological Specimens

**Published:** 1902-09

**Authors:** C. E. S. Flemming


### NOTES ON THREE PATHOLOGICAL SPECIMENS.

BY

C. E. S. Flemming, M.R.C.S., L.R.C.P.

These interesting specimens were shown at a meeting of the Bath and Bristol branch of the British Medical Association on November 27th, 1901.

Fig. 1 is the photograph of a heart with the pulmonary artery perforated by a needle. On a Monday morning I was called to see a child, aged 6, who was said to be dying. I found him dead, and was told the following history. The boy had appeared quite well until the previous Saturday, when, soon after an attack of vomiting, supposed to be due to eating apples, he fell back faint; the fit lasted some minutes, but he soon got all right again. On Monday morning he complained of no pain, ate a good breakfast, ran to the door to get the milk, came back and put it on the table. The mother had meanwhile left the room; when she returned in a few minutes, she found the boy in the next room, lying on the floor, dying or dead.

I next day made a *post-mortem* examination, and on turning back the sternum found the broken end of a fine sewing needle, in the position of the 2nd space just to the left of the sternum: the needle was fairly firmly fixed at the broken end; it passed through the lower edge of the thymus gland. The pericardium was distended and found to be filled with dark fluid blood. The heart was firmly contracted, with all its cavities empty. On turning back the pericardium the point of the needle was found projecting some 2 mm. through it; there was an irregular superficial wound on the front of the pulmonary artery, some 8 mm. long by 2-4 mm. wide, with a complete linear perforation in the centre, as the photograph shows. There was no sign here of any attempt at repair, though inside the artery just at this spot there was a small, thin, ribbon-like *ante-mortem* clot. There was considerable thickness of the parietal pericardium for some distance round the needle, and a few mm. to the right of the needle a small scar as if it had previously been perforated there; on the front of the aorta was a small, round, dark-coloured swelling, looking like a minute aneurysm—it shows as a white spot in the photograph. The fragment of needle was 13 mm. in length. When I told the mother what I had found, she remembered that two months previously the boy on returning from school had said that another child had pricked him in the chest with a needle. She and her husband looked, but saw no mark, and nothing more was thought of the incident.

Evidently when the needle was thrust in it broke and became fixed in the firm tissues between the costal cartilages, and at first lay pointing down to the right, and so, although at the moment of impact it may have injured the aorta sufficiently to cause the small swelling found there, it afterwards receded and remained clear of both vessels. From the recent character of the wound of the pulmonary artery and from the thickening around the needle in the parietal pericardium, it is fairly evident that the needle-point frequently changed its position within this thickened area, and probably did not again enter the pericardial cavity until pushed in by the attack of vomiting. It is remarkable that the fainting attack following this was the only symptom from the day of the accident until the fatal attack; even during the last forty-eight hours, when with probably every respiratory movement damage was being done, there was apparently no marked sign or symptom. Of course one must remember that among this, the labouring class of people, where children are often too much a burden, small signs and symptoms are not noticed, and casual complaints without signs are but little heeded.

Fig. 2 is a photo-micrograph of a section of a small cavernous angioma removed last August from the finger of a woman, aged 23. The patient last June year received a blow from the head of a nail on the outer side of the first phalangeal joint of her forefinger; the skin was not broken, but a painful bruise-like swelling quickly formed, and this prevented her from working for a month. The swelling gradually diminished to the size of a lentil; and this little dark tumour remained unaltered until about six weeks before I removed it, when it began to grow and to be painful. When I saw it it was about 1 c.m. in diameter, moveable under the skin which was not involved, and not altered by pressure; it grew in the not very vascular fibrous tissue at the side and back of the joint, and was easily dissected out—an irregular-shaped, distinctly encapsuled tumour; on section it was dark-red and soft, but apparently solid. I thought it might be a sarcoma, but histologically it proved to be a typical cavernous angioma.

With a history so clear, there can be little doubt that this growth arose from the blood-clot produced by the blow with a nail more than a year before. It is most improbable that there was already a nævus unnoticed in this very spot. This admitted, it is a fact of considerable interest, apart from its unusual occurrence, as possibly throwing some light on the origin of these tumours, and supporting Unna's theory that congenital angiomata are due to intra-uterine pressure, though,

**Fig. 1.**

*Heart—Pulmonary artery perforated by a needle.*

Fig. 2.

*Cavernous angioma of finger.*

Fig. 3.

*Meningeal clot, three years old.*

as I read it, he only applied his theory to the cutaneous variety. Yet as to why one organised and encapsuled clot should disappear and another grow into a distinct tumour there is nothing to show. Histologically there is but little, often no difference between the structure of the vascular highly-organised clot one finds, say, in an old thrombosed common iliac vein and that of a cavernous angioma.

Fig. 3 is from a meningeal clot, judged from the clinical history to be three years old. The clot was, when removed, colourless, and on section its endothelial lined spaces were found to contain no blood cells, but only a hyaline or granular material. How easily one can imagine these spaces, if suddenly connected with some blood supply, growing larger, some coalescing, the whole mass increasing in size without any fresh hemorrhage to account for its growth. Possibly some of the large fleshy meningeal clots of old standing may actually have the life-history, if not the name, of angioma. It would seem as if the blood-clot required to be encapsuled in a position poorly supplied with blood-vessels, so that it should not be absorbed, while it evolved in itself some condition which under some fresh circumstance, *e.g.* the ingrowth of a blood-vessel, should start it growing.

Rokitansky's theory is that the connective tissue element of an angioma is developed outside the vascular system, and only connected at a later date. This can quite accord with the history of my case, where probably for many months the organised clot remained like the meningeal clot (Fig. 3) until its endothelial lined spaces became in some way directly connected with a blood supply; but still, as Rokitansky implies, the connective tissue must contain some element of new growth, either from inclusion in the original clot at the time of its formation or from some personal factor, or from both combined.

I have to thank Mr. James Taylor, of Clifton, for very kindly preparing the excellent photomicrographs.

## Figures and Tables

**Fig. 1. f1:**
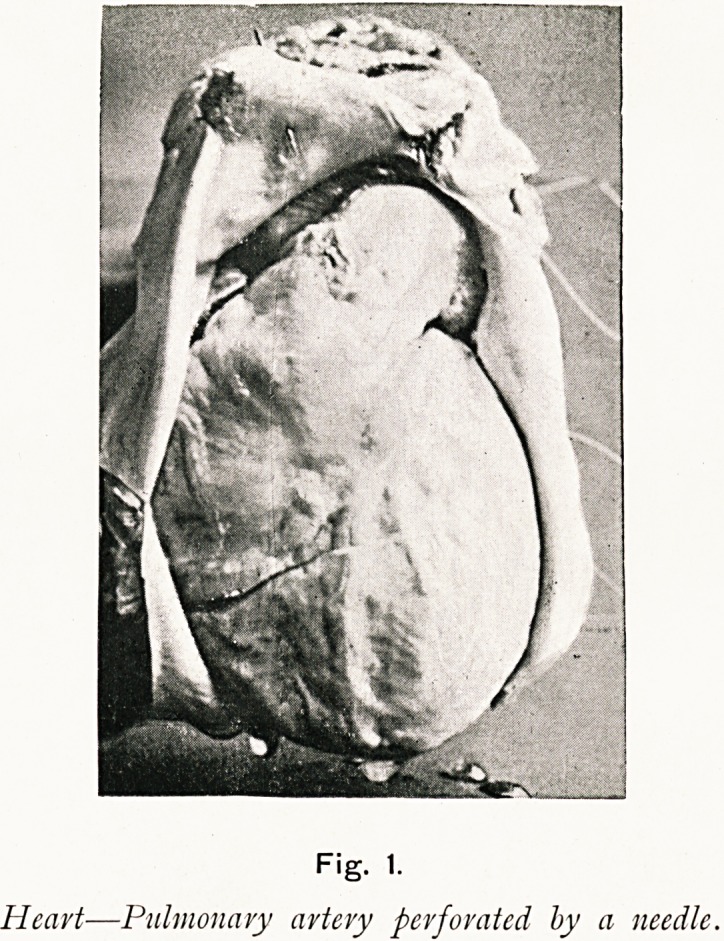


**Fig. 2. f2:**
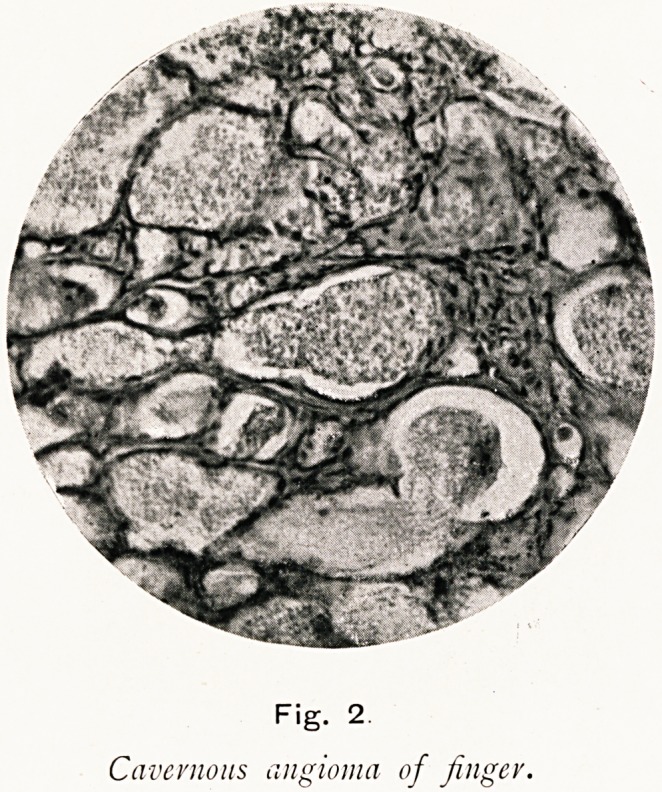


**Fig. 3. f3:**